# Re-assess Vector Indices Threshold as an Early Warning Tool for Predicting Dengue Epidemic in a Dengue Non-endemic Country

**DOI:** 10.1371/journal.pntd.0004043

**Published:** 2015-09-14

**Authors:** Fong-Shue Chang, Yao-Ting Tseng, Pi-Shan Hsu, Chaur-Dong Chen, Ie-Bin Lian, Day-Yu Chao

**Affiliations:** 1 Graduate Institute of Microbiology and Public Health, College of Veterinary Medicine, National Chung-Hsing University, Taichung, Taiwan; 2 Graduate Institute of Statistics and Information Science, National Changhua University of Education, Changhua, Taiwan; 3 Department of Family Medicine, Taichung Hospital, Department of Health, Executive Yuan, Taiwan, R.O.C; 4 Department of Health, Kaohsiung City Government, Kaohsiung City, Taiwan; Santa Fe Institute, UNITED STATES

## Abstract

**Background:**

Despite dengue dynamics being driven by complex interactions between human hosts, mosquito vectors and viruses that are influenced by climate factors, an operational model that will enable health authorities to anticipate the outbreak risk in a dengue non-endemic area has not been developed. The objectives of this study were to evaluate the temporal relationship between meteorological variables, entomological surveillance indices and confirmed dengue cases; and to establish the threshold for entomological surveillance indices including three mosquito larval indices [Breteau (BI), Container (CI) and House indices (HI)] and one adult index (AI) as an early warning tool for dengue epidemic.

**Methodology/Principal Findings:**

Epidemiological, entomological and meteorological data were analyzed from 2005 to 2012 in Kaohsiung City, Taiwan. The successive waves of dengue outbreaks with different magnitudes were recorded in Kaohsiung City, and involved a dominant serotype during each epidemic. The annual indigenous dengue cases usually started from May to June and reached a peak in October to November. Vector data from 2005–2012 showed that the peak of the adult mosquito population was followed by a peak in the corresponding dengue activity with a lag period of 1–2 months. Therefore, we focused the analysis on the data from May to December and the high risk district, where the inspection of the immature and mature mosquitoes was carried out on a weekly basis and about 97.9% dengue cases occurred. The two-stage model was utilized here to estimate the risk and time-lag effect of annual dengue outbreaks in Taiwan. First, Poisson regression was used to select the optimal subset of variables and time-lags for predicting the number of dengue cases, and the final results of the multivariate analysis were selected based on the smallest AIC value. Next, each vector index models with selected variables were subjected to multiple logistic regression models to examine the accuracy of predicting the occurrence of dengue cases. The results suggested that Model-AI, BI, CI and HI predicted the occurrence of dengue cases with 83.8, 87.8, 88.3 and 88.4% accuracy, respectively. The predicting threshold based on individual Model-AI, BI, CI and HI was 0.97, 1.16, 1.79 and 0.997, respectively.

**Conclusion/Significance:**

There was little evidence of quantifiable association among vector indices, meteorological factors and dengue transmission that could reliably be used for outbreak prediction. Our study here provided the proof-of-concept of how to search for the optimal model and determine the threshold for dengue epidemics. Since those factors used for prediction varied, depending on the ecology and herd immunity level under different geological areas, different thresholds may be developed for different countries using a similar structure of the two-stage model.

## Author Summary

With the continuously high levels of worldwide dengue transmission, predicting dengue outbreaks in advance of their occurrence or identifying specific locations where outbreak risks are highest is of critical importance. However, only few studies have been conducted in dengue non-endemic countries to evaluate the association of vector index with the occurrence of dengue cases; and the establishment of an early warning signal would significantly enhance the public health intervention. Our study here provided the proof-of-concept results, utilizing a two-stage model to identify the best set of lag effects of meteorological and entomological variables, explaining dengue epidemics based on the data obtained from Taiwan, which is a dengue-non-endemic country. Each of the vector indices when combined with the meteorological factors has better performance compared to the prediction using AI, BI, CI and HI alone, with 83.8, 87.8, 88.3 and 88.4% accuracy, respectively. Because of the complex interplays between the size of human hosts and movement, environmental factors and dynamic changes of mosquito population and density, each country should consider its own individual data and situation and apply this two-stage model to find the optimal predictive models for allocating public health resources and prevention strategies.

## Introduction

Dengue viruses (DENV) are the most widespread arthropod-borne viruses affecting humans. A recent study estimates that annually 390 million DENV infections occur worldwide with 500,000 severe cases and 25,000 deaths, mostly affecting children[1]. Infection with DENV can result in a range of outcomes from asymptomatic infection to clinical manifestations ranging from dengue fever (DF) to the life threatening complications of dengue hemorrhagic fever (DHF) and shock syndrome (DSS). This mosquito-borne disease is caused by four serotypes of dengue virus (DENV-1 to 4), which belong to the family *Flaviviridae*, genus *Flavivirus*[2]. Infection by one serotype of DENV will provide lifelong immunity to that particular strain but not to the remaining three serotypes, which usually lead to the reduction of the susceptible population. However, immunity from prior infection might enhance the incidence of DHF through antibody-dependent enhancement mechanism even though the transmission of DENV is reduced[3,4]. The virus is transmitted to humans mainly by two mosquito vectors, *Aedes aegypti* or *Aedes albopictus*. In the absence of an effective vaccine or specific therapy, vector control remains the only way to prevent dengue viral transmission[5].

Increased travel with population movement, global trade, crowded urban living conditions, global warming, virus evolution and ineffective vector-control strategies are also increasing the risk of dengue transmission in the world[6,7]. Travelers infected with dengue virus during their trip returning home may place the local population at risk wherever mosquito vectors are present[8,9]. Therefore, the required conditions for the occurrence of a dengue outbreak in countries where dengue is not endemic include i) the presence of dengue viruses through repeated introduction of imported cases, ii) a sufficient density of competent vectors above the threshold, iii) a sufficient number of susceptible population, and iv) a favorable climatic and environmental condition for dengue transmission[10]. Furthermore, numerous studies suggested an effect of climate on DENV transmission through changes in vector population size and distribution. The relationships between entomological measures of risk and human infection are not well understood[11–13].

The mosquito vectors, principally *A*. *aegypti*, become infected when they feed on humans during the usual five-day period of viraemia. The virus passes from the mosquito intestinal tract to the salivary glands after an extrinsic incubation period, a process that takes approximately 10 days, which may vary depending on the ambient temperatures[14]. Mosquito bites after the extrinsic incubation period result in infection, which might be promoted by mosquito salivary proteins[15–17]. The abundance of dengue vector as well as dengue transmission generally exhibits seasonal variation depending on the local ecology and urban environment. Therefore, vector surveillance is recommended by the World Health Organization (WHO) and is a routine practice in many dengue-occurring countries to provide quantifiable measure of fluctuations in magnitude and geographical distribution of dengue vector populations[18,19]. The traditional standard protocol relies on surveys of larvae and pupae, which include three most commonly used indices: the House index (HI), the Container index (CI) and Breteau index (BI). A poor correlation with the abundance of adult mosquitoes has caused their sensitivity and reliability to be questioned[20,21]. The alternative, pupal indices developed by Focks et al[22], has been suggested to better reflect the risk for transmission, but the utility for source reduction programs is still controversial[23,24]. The most accurate method of vector surveillance is the capture of adult mosquitoes by aspiration, which directly counts dengue vectors that are actively in search of a blood meal: adult female *A*. *aegypti* and occasionally *A*. *albopictus* mosquitoes. However, capturing adult mosquitoes is labor-intensive, and requires access to premises. Recently, fixed-position traps, designed to capture gravid mosquitoes using water-filled pots in which *A*. *aegypti* lay their eggs, are widely used as a simple sampling tool[25,26]. However, its correlation with the incidence of dengue is still controversial[27,28].

Kaohsiung City, a modern metropolis of 1.5 million people, has been afflicted by different serotypes of DENV and has become the focus of dengue virus activity in Taiwan during the recent decades[29]. During 2002–2011, Kaohsiung City had annual outbreaks of variable scales, resulting in more than 6,000 confirmed cases[30]. Since 2005, vector surveillance activities by the Department of Health, Kaohsiung City Government, were initiated by using specially trained personnel. Four different vector indices were chronically established. A previous study suggested that adult *Aedes* mosquito index from 2005–2009 showed temporal correlation with the peak of the DF activity with a lag period of 1–2 months[29]. However, the association between different vector indices and the occurrence of dengue cases has not been comprehensively evaluated. Therefore, the objectives of this study were to i) evaluate the temporal relationship between meteorological variables, entomological surveillance indices and dengue confirmed cases, ii) identify the suitable conditions for an epidemic occurrence, and iii) establish the threshold for entomological surveillance indices as an early warning tool for dengue epidemic.

## Materials and Methods

### Study area

**Fig 1:**
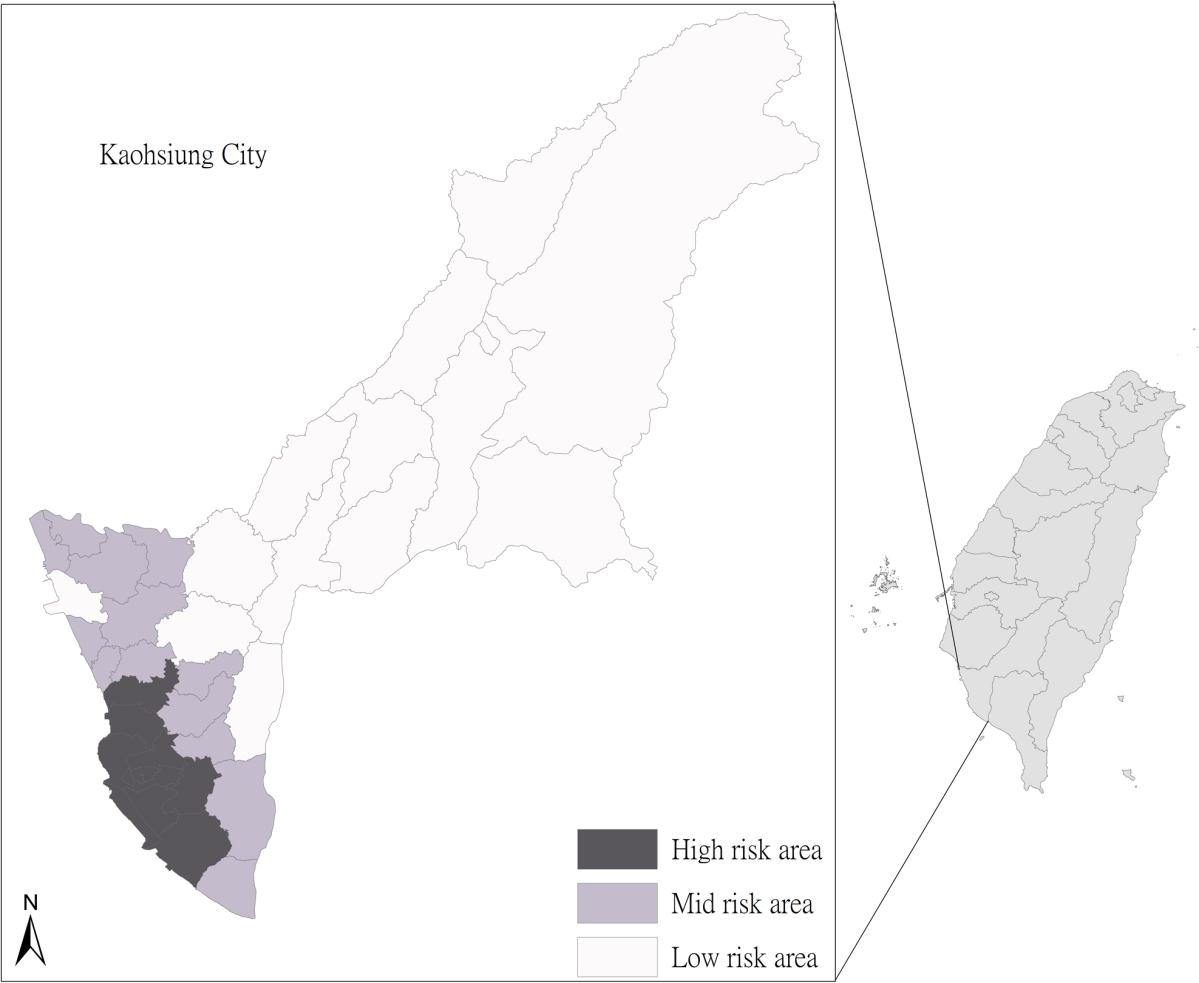
The location of Kaohsiung city in Taiwan. The inset shows the 38 districts, including 11 districts from the old Kaohsiung administrative districts. All districts were further classified into high, middle (mid) and low risk areas based on the household density and the average number of households with the presence of *A*. *aegypti* from the historical entomological data.

Although dengue virus epidemics have occurred annually in Taiwan for the past decade, the main focus of activity has been in Kaohsiung City (Fig 1). Kaohsiung City is a standard subtropical region with annual average rainfall from 1796.7 to 2821.4 mm and concentrated from May to September. In addition, the annual average temperature is from 24.9 to 25.7 degrees Celsius (°C), with the lowest average 11.6°C in February and the highest average 31.5°C in June. After December 25, 2010, the area of Kaohsiung city expanded due to the combined administration area between Kaohsiung County and Kaohsiung City. Since our study period covered from January 2005 to December 2012, the study area included the former Kaohsiung City, Fongshan, Daliao, and Linyuan districts as well as the adjacent Pingtung County and Tainan City in southern Taiwan, located between 120°10′32″ to 121°01′15″ east longitudes and 22°28′ to 23°28′ north latitudes.

### Data collection

#### Meteorological data

We systematically collected daily weather data for Kaohsiung City that was publicly available through the 9 branch stations of the Environmental Protection Administration (EPA). Due to the strong co-linearity among the daily minimum, maximum and average of the meteorological data, only one type of daily data could be used in the model. Through the two-stage model examination with the largest sum of sensitivity and specificity under the criteria of selecting the predicting threshold (detailed explanation in the following statistical analysis section), the meteorological variables finally analyzed in this study included the daily accumulative rainfall, daily mean relative humidity and daily mean temperature. Also, the nonlinear effect of the meteorological variables on the dengue case counts was noted after examining the raw data (S1 Text). Therefore, the entire meteorological datum was trisected into three levels (low, medium and high) according to the 33^rd^ and 66^th^ percentile.

#### Dengue case surveillance data

Dengue is classified as a reportable infectious disease and suspected cases must be reported within 24 hours for a clinical diagnosis in Taiwan. Cases of ‘‘probable DF” are patients with body temperatures >38^°^C and two or more of the following clinical manifestations: headache, retro-orbital pain, myalgia, arthralgia, rash, hemorrhagic manifestations and leucopenia. The dengue case surveillance system in Taiwan is made up of two parts: active and passive surveillance for the comprehensive and effective surveillance of dengue infection[31]. The active surveillance includes fever screening at the airport (identifying fever cases by infrared thermal scanner, which has been routinely operated by the government since 2003), and health statements from the inbound passengers[32,33]. To reinforce the surveillance system, once confirmed dengue cases are identified, the epidemiological investigation will be undertaken around the residential areas, schools, and work places; and specimens of febrile cases are taken as part of the active surveillance. The passive surveillance refers to the hospital-based reporting system for the notification of either imported or domestic dengue cases. The serum specimens from the suspected dengue patients are sent to the central laboratory for laboratory confirmation. The laboratory confirmation of dengue includes nucleic acid identification of dengue virus by reverse-transcriptase polymerase chain reaction (RT-PCR), serological testing on single or paired serum samples by dengue-specific envelope and membrane–specific immunoglobulin M (IgM) and IgG antibody-capture enzyme-linked immunosorbent assay (with the exclusion of Japanese encephalitis virus infection), or virus isolation[34]. All relevant data and diagnostic results are reported via the web-based National Surveillance System for subsequent tracking and management[35].

The analyses in this study used data from the confirmed dengue cases obtained from the National Notifiable Disease Surveillance System of the Taiwan Centers for Disease Control (Taiwan-CDC) and included the date of ascertainment, residency (detailed to “Li”, the smallest administrative units), country in which infection was acquired, age at diagnosis and gender. The definition of a confirmed dengue case includes the positive detection of RNA or viruses, IgM titer positivity or four-fold rises of IgG titer by laboratory diagnoses[32]. A domestic or indigenous dengue case was considered a confirmed case in which the patient had not traveled abroad within two weeks prior to the onset of illness.

#### Vector surveillance data

Vector surveillance activities by the Department of Health (DOH), Kaohsiung City Government, were initiated in 2005 by specially trained personnel. All personnel had received training in mosquito species distinction, mosquito habitat recognition techniques and sampling methods. The Li was used as the surveying unit in which 50–100 households were randomly selected for inspection of larval habitats or infestation of *A*. *aegypti* and *A*. *albopictus* mosquitoes under the guidelines recommended by the World Health Organization (WHO)[19]. The inspection frequency of each Li was based on risk level according to household density and the average number of households with the presence of *A*. *aegypti* based on the previous entomological data. If the household density is higher than 1,000 households per square kilometer and the prior annual average of BI is higher than 4, those Lis are classified as the high risk district. If the household density is between 260–1,000 households per square kilometer and the prior annual average of BI is lower than 4 but higher than 3, those Lis are classified as the middle risk district. The rest of Lis are classified as the low risk district. Each Li was visited for vector inspection, which covered indoor and outdoor areas of the selected premise, on a weekly, monthly and bi-monthly basis depending on the high, middle and low risk districts, respectively (Fig 1). Adult *Aedes* mosquitoes were captured indoors and outdoors with hand-nets at 8:30–11:30 AM or 1:30–4:30 PM. The captured adult mosquitoes were further identified as *A*. *aegypti* or *A*. *albopictus* and the numbers were recorded accordingly. Capture activities were completed for all rooms, including the basement, within a maximum of 10 minutes for each inspected premise. Containers with immature *Aedes* mosquitoes (larvae/pupae) were considered as positive containers. For habitats with low water volume (<30 liter) the larvae/pupae were strained off and transferred into white bowls for visualization and counting. For habitats containing high water volume, as many larvae/pupae were collected as possible and the mosquito species was determined following adult emergence from the collected specimens reared at the laboratory facilities of the DOH, Kaohsiung city[36].

In this study, we focused on the high risk district where the inspection was carried on a weekly basis and 97.9% dengue cases occurred. Three mosquito larval indices (Breteau, Container and House indices) were used to estimate the density of immature *Aedes* mosquitoes in the study. The BI was defined as number of positive containers per 100 houses. The CI was calculated as percentage of water-holding containers infested with larvae or pupae. The HI was calculated as the number of houses with at least one larval breeding site positive for *A*. *aegypti* divided by the number of inspected premises. The adult index (AI) was calculated as the number of adult female mosquitoes captured divided by the number of inspected premises. Since the number of *A*. *aegypti* captured was much more abundant than those of *A*. *albopictus* and the positive correlation between both numbers was observed (p<0.05), only the number of *A*. *aegypti* was used to calculate the AI.

### Ethics statement

This study was approved by the Institutional Review Board (Approval No. IRB-R-05-002) of Taichung Hospital, Ministry of Health and Welfare, Taiwan; and all analyzed data was anonymized.

### Statistical analysis

Since Taiwan is not a dengue-endemic country, the common season for the indigenous dengue cases to occur starts from May to December after repeated introduction of imported dengue cases as suggested by previous publication[8]. The dengue cases within this period comprised 98.7% of the annual total. Therefore, we focused our forecasting model from May to December. We used a two-week interval as a unit to divide the 8-year span into 151 intervals, counting the dengue case (Y) and averaged the meteorological data. The environmental factors included in the study were vector indices (VI, including AI, BI, CI, and HI), mean temperature (Temp,°C), mean rainfall (RF, mm) and relative humidity (RH, %). We calculated the mean of the daily average over each week in the study period for all the weather factors, so that the corresponding 33^rd^ and 67^th^ percentiles can be determined. We then further transformed these factors into indicator variables of three levels (low, medium, and high) by using the percentiles as the cutoffs.

Besides, both 2-week and 1-month lags of VI and meteorological factors were considered here. The 2-week lag took into consideration the blood feeding of mosquito on an undetected viremic subject and the 7–10 days interval to be able to re-infect a new subject, who requires 3–5 days to be symptomatic once infected [37]. On the other hand, the 1-month lag took into consideration the mosquito life cycle from the laying to hatching of eggs, which requires 2 weeks and another 2 weeks from feeding to infect a new subject. Therefore, the VI distinguishes into VI_1_ (2-week lag) and VI_2_ (1-month lag), and each VI_1_ or VI_2_ was also calculated separately based on individual AI, BI, CI, and HI. To avoid colinearity among the VIs, we considered only one of the VIs at a time (e.g., AI 2-week lag) joined by 6-weather variables (RF_1_ (2-week lag), RF_2_ (1-month lag), Temp_1_ (2-week lag), Temp_2_ (1-month lag), RH_1_ (2-week lag), RH_2_ (1-month lag) as potential predicting variables. We used the minimized Akaike’s information criteria (AIC) as the criterion for models selection, and each variable was either included or excluded; and therefore a total of 2^7^ = 128 combinations were tried to select the optimal subset of predicting variables. Since there were 4 vector indices with 2 lag options, a total of 128×4×2 models were tried.

Regression models were developed by using a two-stage approach, wherein we first performed an exploratory analysis to select the best models and used the selected model to create the indices for predicting the occurrence of dengue cases and then estimated the prediction accuracy. The stage 1 of the initial exploratory analysis used the Poisson regression to select the optimal subset of variables and time-lags for predicting the number of DF cases. The stage 2 used the optimized model selected from Stage 1 to establish the prediction threshold and defined the prediction accuracy from the ROC curve by logistic regression.

In stage 1, the univariate and multivariate lagged-time Poisson regression analysis was performed to assess the relationship between the environmental factors and dengue cases. A basic multivariate Poisson regression model was written as below.

$$\begin{array}{l} Y\sim Poisson(\lambda ),\\ \text{Ln}(\lambda )=\beta_{0}+\beta_{1}\cdot VI_{1}+\beta_{2}\cdot VI_{2}+\beta_{3}\cdot RF_{11}+\beta_{4}\cdot RF_{12}+\beta_{5}\cdot RF_{21}+\beta_{6}\cdot RF_{22}\\ \;+\beta_{7}\cdot Temp_{11}+\beta_{8}\cdot Temp_{12}+\beta_{9}\cdot Temp_{21}+\beta_{10}\cdot Temp_{22}\\ \;+\beta_{11}\cdot RH_{11}+\beta_{12}\cdot RH_{12}+\beta_{13}\cdot RH_{21}+\beta_{14}\cdot RH_{22} \end{array}$$

where Y is the incidence of confirmed dengue cases and β_0_ is the intercept. VI_1_ and VI_2_ are indicator variables with value 1 if the 2-week lag and 1-month lag, respectively, are above the (overall) 33^rd^ percentile; and 0 otherwise. RF_11_, Temp_11_, and RH_11_ are indicator variables with value 1 if the RF_1_, Temp_1_ and RH_1_, respectively, are between the (overall) 33^rd^ and 67^th^ percentiles; and 0 otherwise. Analogously, RF_12_, Temp_12_ and RH_12_ are value 1 if the RF_1_, Temp_1_ and RH_1_, respectively, are above the 67^th^ percentiles. In addition, RF_21_, Temp_21_ and RH_21_ are indicator variables with value 1 if the RF_2_, Temp_2_ and RH_2_, respectively, are between the (overall) 33^rd^ and 67^th^ percentiles; and 0 otherwise. Similarly, RF_22_, Temp_22_ and RH_22_ are value 1 if the RF_2_, Temp_2_ and RH_2_, respectively, are above the 67^th^ percentiles.

In stage 2, we fitted Y on the 4 optimal subset of variables selected from stage 1, and produced the ROC curves. We considered count of outbreak for bi-week greater than 1, then definition of binary variable Y = 1 and = 0 if not. The final optimal models selected were variables including VI_2_, RF_1_, RF_2_, Temp_1_, and RH_1_ and the predicting coefficients for each VI were as below. For each ROC curves, we picked a point that looked farthest from the diagonal line, which has the largest area under curve (AUC) and with maximum total of sensitivity and specificity. The value decided by that point was then used as the predicting threshold. We defined the corresponding linear combinations of variables estimated from the logistic regression as a prediction index and the thresholds for each VI were calculated accordingly. As a result, there were 4 models for each index: AI, BI, CI, and HI.

### Sensitivity analysis

We conducted 2 cross-validations to examine the sensitivity of our method. The first is the leave-one-out approach, of which each observation (a two-week period) was removed and the rest were used to establish the classification criterion via multiple logistic regression and corresponding AUC, and then that criterion was used to classify the removed one. After iterating on all the periods, the performance was assessed by the average accuracy rate. The second approach was to leave one-year out instead of one period[38]. We repeated the above process on Model-AI, Model-BI, Model-CI and Model-HI separately.

Two-tailed p<0.05 was regarded as statistically significant. The lagged-time Poisson regression analyses were performed by using SAS Version 9.3 for Windows (SAS Institute Inc., Cary, North Carolina, USA).

## Results

### Temporal trend of dengue cases and meteorological data

**Fig 2:**
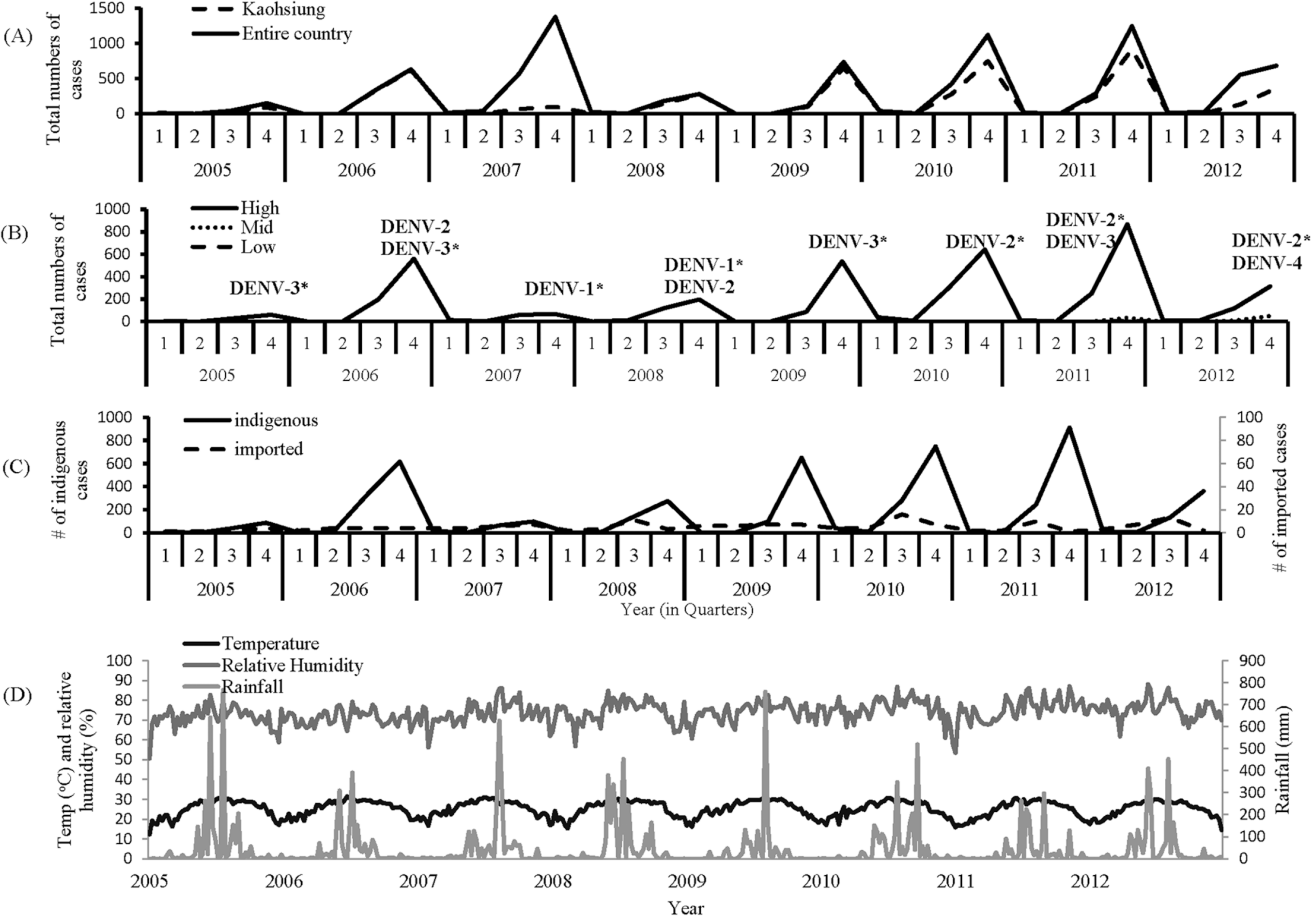
Secular trend of the meteorological data and the dengue cases from 2005 to 2012. (A) Comparison between Kaohsiung city and whole Taiwan of all laboratory-confirmed indigenous dengue cases from 2005 to 2012 based on the residential area. (B) Comparison among high, middle and low risk areas of all laboratory-confirmed indigenous dengue cases from 2005 to 2012. All dengue virus serotypes detected during each epidemic was indicated accordingly, with the dominant serotype labeled with asterisk based on the major serotype detected from more than 80% of dengue cases in the specific year. (C) The quarterly total numbers of the laboratory-confirmed imported and indigenous dengue cases in Kaohsiung city from 2005 to 2012. (D) The weekly average of temperature (temp, ^o^C), rainfall (rain, mmHg) and relative humidity (rh, %) from 2005 to 2012.

From January 2005 to December 2012, Taiwan-CDC recorded 8,918 laboratory-confirmed cases of dengue virus infections in Taiwan, and 58.1% were from Kaohsiung City (Fig 2A). The cases were detected by passive and active surveillance activities. The successive waves of dengue outbreaks with different magnitudes were recorded in Kaohsiung City, and they involved a dominant serotype of DENV during each epidemic, representing more than 80% of cases confirmed by virus detection in the specific year (Fig 2B). The annual dengue incidence rate varied with the highest rate (1,176 cases) observed in 2011caused by DENV-2 and DENV-3; and the lowest rate (102 cases) in 2005 caused by DENV-3 without significantly secular trend. By dividing the annual dengue cases into four quarters of the year, the annual outbreak usually started one month before the third quarter and reached a peak in the fourth quarter. The dengue cases during the first quarter were residual cases from the outbreak in the previous year. Based on the geographical distribution, 98, 1.9 and 0.2% of dengue cases were from the high, middle and low risk districts, respectively (Fig 2B). From 2005 to 2012, 159 total dengue cases were confirmed as imported, which distributed evenly throughout different months without a secular trend (Fig 2C). The peaks of the confirmed cases detected by passive and active surveillance were also coincided as shown in S1 Fig.

The average temperature in Kaohsiung City is around 25^°^C with the hottest season occurring in July to September and the coldest in January to March. The hottest season usually coincided with the strike of tropical hurricanes, which brought in significant amount of rainfall, causing a rise in the annual dengue epidemic (Fig 2D). The relative humidity was relatively stable with an annual average of around 72.4%.

### Seasonal trend of entomological data

**Fig 3:**
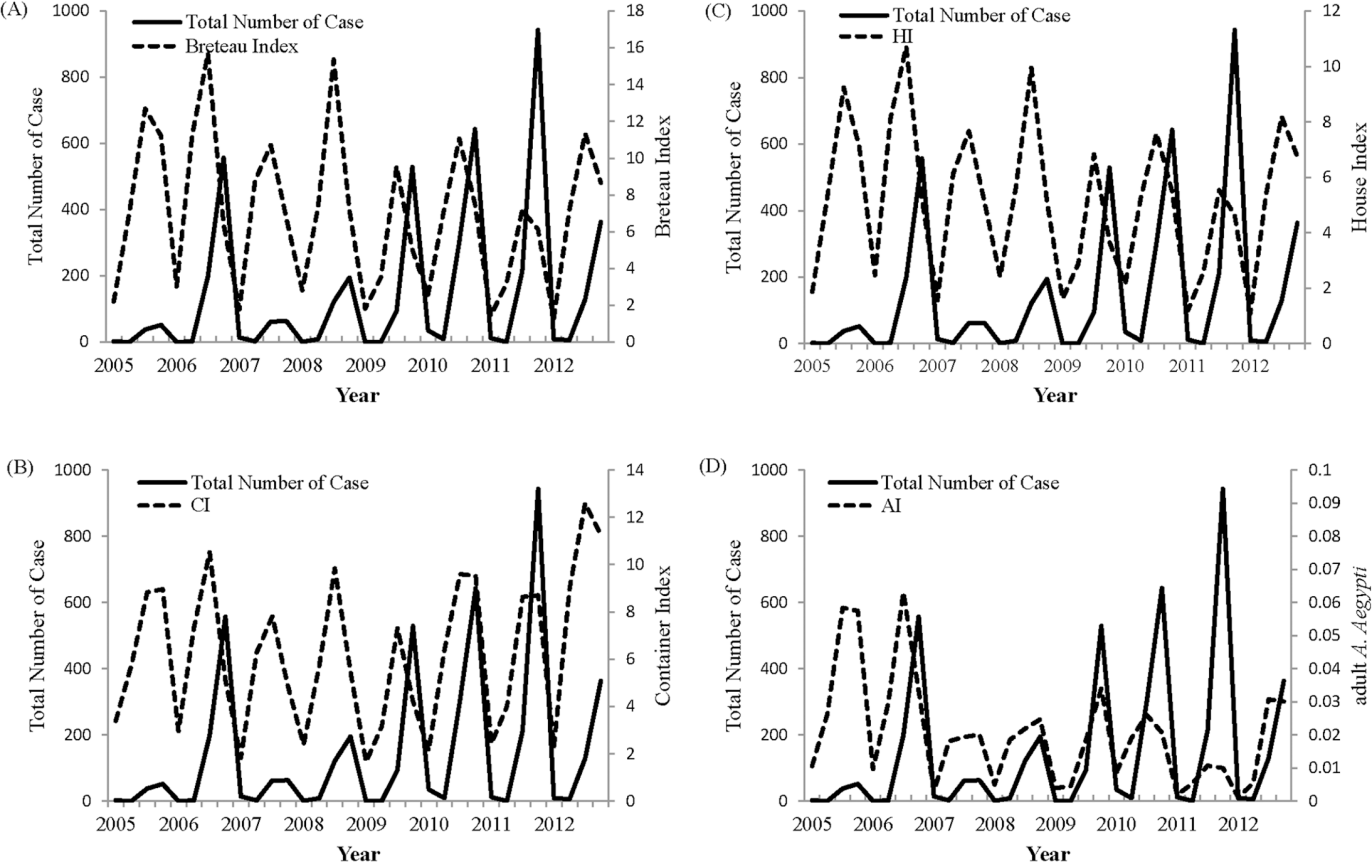
The temporal relationship between the indigenous dengue cases and the vector indices from the entomological surveillance data from 2005 to 2012 including Breteau index (A), Container index (B), House index (C) and adult *A*. *aegypti* index (D).

The density of *A*. *aegypti* and *A*. *albopictus* in Kaohsiung City showed dynamic and periodic variation. We focused only on the high risk district for data analysis wherein about 97.9% dengue cases occurred and the inspection of immature and mature mosquitoes was carried out on a weekly basis. BI, CI or HI over 5 usually appeared in early summer and peaked during autumn in the high risk area (Fig 3). Vector data from 2005–2012 showed that the peak of the adult mosquito population was followed by a peak in the corresponding dengue activity with a lag period of 1–2 months (Fig 3). Since Kaohsiung City frequently had more dengue epidemics, which occurred annually, and had a more comprehensive vector surveillance data, the following statistical analyses was focused on the data from Kaohsiung City.

### Univariate Poisson analysis of lag-effect

Results of univariate analysis showed that the risk of an increased number of dengue cases was significantly associated with the increase in all vector indices (including BI, CI, HI and AI) on either 2-week- or 1-month-lag effect as shown in Table 1. However, the meteorological variables showed different patterns of association of dengue epidemics. Either medium or high level of the temperature showed negative association with the increased risk of dengue epidemics at 2-week-lag effect. However, the medium temperature showed a positive association with the increased risk (RR: 1.32; 95% CI: 1.23–1.41), but the high level temperature showed a negative association (RR: 0.77; 95% CI: 0.71–0.83) at 1-month lag with statistical significance (p<0.05). Similarly, both medium and high levels of RF showed negative associations with the increased risk of dengue epidemics at 2-week-lag effect. However, the medium RF showed positive association with increased risk (RR: 1.12; 95% CI: 1.05–1.2), but the high level temperature showed a negative association (RR: 0.86; 95% CI: 0.80–0.92) at 1-month lag. The increase of RH showed consistently strong correlation with the increased risk of dengue cases either at 2-week- or 1-month-lag effect (Table 1).

**Table 1:** Univariate analysis of risk factors for dengue incidence by Poisson regression model.

Risk factor	stratification	Risk	95% CI	p-value
**2-week lag**				
Breteau index	medium	1.81	(1.68, 1.95)	<0.0001
Breteau index	high	1.39	(1.29, 1.51)	<0.0001
Container index	medium	2.04	(1.87, 2.23)	<0.0001
Container index	high	3.01	(2.77, 3.27)	<0.0001
Adult female mosq index	medium	1.46	(1.34, 1.58)	<0.0001
Adult female mosq index	high	1.98	(1.83, 2.13)	<0.0001
House index	medium	1.51	(1.41, 1.63)	<0.0001
House index	high	1.21	(1.12, 1.31)	<0.0001
Larva index	medium	1.49	(1.38, 1.6)	<0.0001
Larva index	high	1.3	(1.21, 1.41)	<0.0001
Temperature	medium	0.705	(0.66, 0.752)	<0.0001
Temperature	high	0.381	(0.351, 0.413)	<0.0001
Rain	medium	0.819	(0.766, 0.875)	<0.0001
Rain	high	0.546	(0.506, 0.59)	<0.0001
Relative humidity	medium	1.08	(1.001, 1.17)	0.048
Relative humidity	high	1.65	(1.53, 1.77)	<0.0001
**1-month lag**				
Breteau index	medium	2.11	(1.95, 2.29)	<0.0001
Breteau index	high	2.12	(1.96, 2.3)	<0.0001
Container index	medium	3.47	(3.13, 3.85)	<0.0001
Container index	high	5.28	(4.78, 5.84)	<0.0001
Adult female mosq index	medium	1.4	(1.3, 1.52)	<0.0001
Adult female mosq index	high	1.97	(1.83, 2.13)	<0.0001
House index	medium	1.84	(1.7, 2)	<0.0001
House index	high	1.87	(1.73, 2.03)	<0.0001
Larva index	medium	2.15	(1.98, 2.33)	<0.0001
Larva index	high	2	(1.85, 2.17)	<0.0001
Temperature	medium	1.32	(1.23, 1.41)	<0.0001
Temperature	high	0.766	(0.709, 0.828)	<0.0001
Rain	medium	1.12	(1.05, 1.2)	0.001
Rain	high	0.856	(0.795, 0.922)	<0.0001
Relative humidity	medium	1.22	(1.12, 1.33)	<0.0001
Relative humidity	high	2.34	(2.17, 2.52)	<0.0001

### Multivariate modeling of dengue outbreak risk

**Fig 4:**
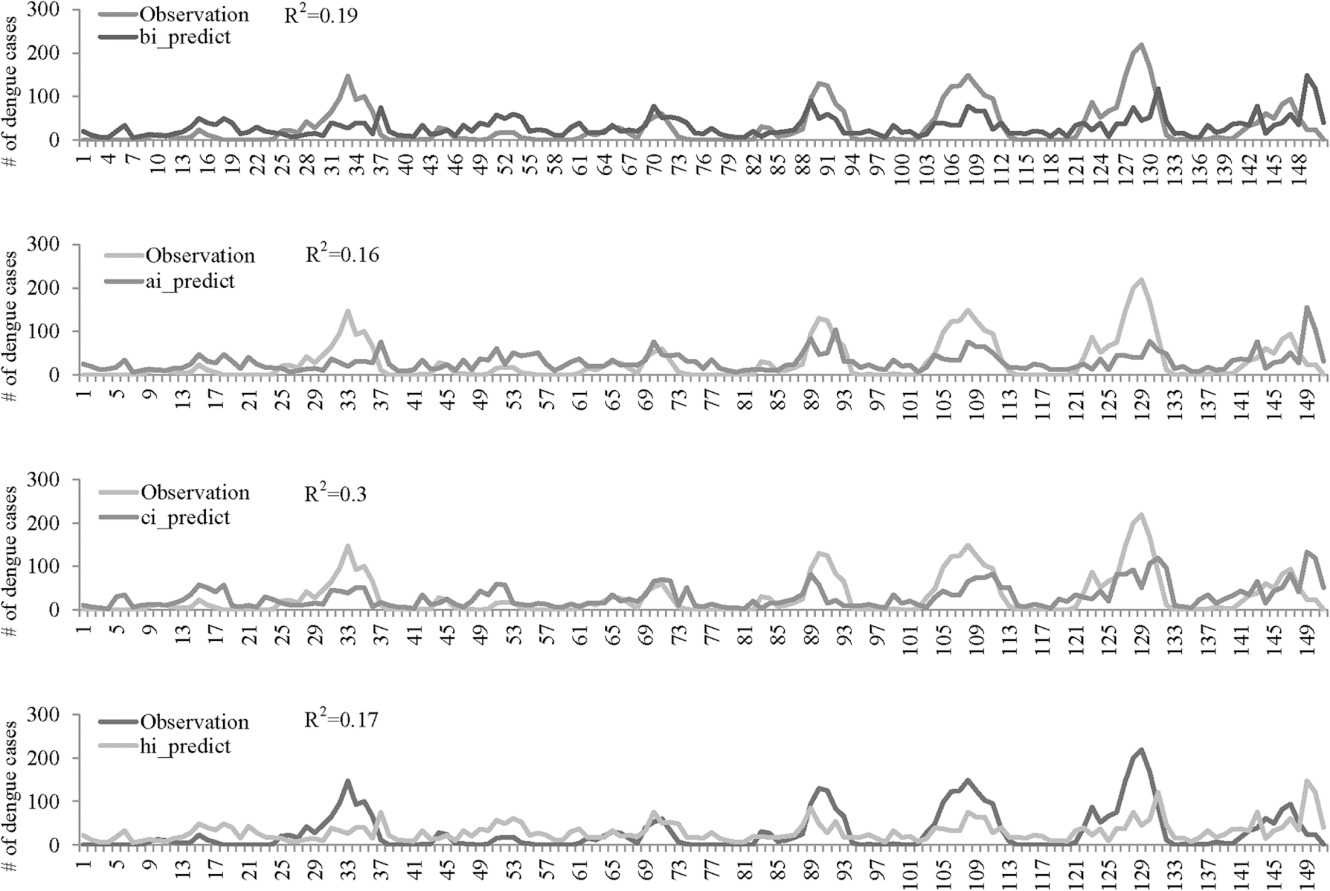
The weekly number of dengue cases from 2005 to 2012 based on the observation (solid line) and prediction (dahs line) from each vector index model including Model-BI: Breteau index model (A), Model-AI: adult *A*. *aegypti* index model (B), Model-CI: Container index model (C) and Model-HI: House index model (D). The values of coefficient of determination (R-square) from each vector index model were also indicated.

In order to find the best model for the prediction of dengue occurrence, a multivariable Poisson regression model was fitted to the data to search for independent factors by running different combinations of time lag effect. The final results of the multivariate analysis that best predicted the occurrence of dengue cases were selected based on the smallest AIC value as shown in Table 2. The 1-month lag effect of all VI was selected in the multivariable Poisson model except AI, for which a 2-week-lag effect showed the best result. The 2-week-lag effect of all meteorological factors was also selected in the final multivariable Poisson model; however, RF and Temp showed a negative association with the occurrence of dengue cases. In contrast, RH and 1-month lag of RF showed a positive association. A slight difference with statistical significance was also noted at Model-CI, in which the 1-month lag of RF showed positive association when the RF level was medium (RR:1.12; 95% CI: 1.04–1.21) and negative association when it was at a high level (RR:0.89; 95% CI: 0.81–0.98). Although the error between the observed and estimated counts was large (range of R-square of four models: 0.16–0.3), the prediction of peaks by the predictors selected from Poisson model quite coincided (Fig 4).

**Table 2:** Multivariate analysis of risk factors for dengue incidence.

variable	risk	estimator	95% CI	risk	95% CI	p-value
**Model-BI**						
BI lag-1-month	medium+high	0.906	(0.828, 0.983)	2.47	(2.29, 2.67)	<.0001
rain lag-2-week	medium	-0.106	(-0.188, -0.025)	0.385	(0.347, 0.428)	0.011
rain lag-2-week	high	-0.953	(-1.06, -0.848)	0.899	(0.829, 0.976)	<.0001
rain lag-1-month	medium	0.228	(0.152, 0.305)	1.26	(1.16, 1.36)	<.0001
rain lag-1-month	high	0.078	(-0.02, 0.177)	1.08	(0.98, 1.19)	0.118
temp lag-2-week	medium	-0.352	(-0.442, -0.261)	0.703	(0.643, 0.77)	<.0001
temp lag-2-week	high	-1.06	(-1.17, -0.961)	0.345	(0.383, 0.345)	<.0001
rh lag-2-week	medium	0.401	(0.319, 0.484)	1.48	(1.38, 1.62)	<.0001
rh lag-2-week	high	1.08	(0.995, 1.16)	2.94	(2.71, 3.2)	<.0001
**Model-CI**						
CI lag-1-month	medium+high	1.68	(1.58, 1.78)	5.35	(4.84, 5.92)	<.0001
rain lag-2-week	medium	-0.193	(-0.274, -0.111)	0.432	(0.389, 0.479)	<.0001
rain lag-2-week	high	-0.84	(-0.945, -0.736)	0.825	(0.76, 0.895)	<.0001
rain lag-1-month	medium	0.115	(0.037, 0.194)	1.12	(1.04, 1.21)	0.004
rain lag-1-month	high	-0.114	(-0.21, -0.018)	0.892	(0.811, 0.982)	0.02
temp lag-2-week	medium	-0.25	(-0.34, -0.159)	0.779	(0.712, 0.853)	<.0001
temp lag-2-week	high	-1.1	(-1.2, -0.993)	0.334	(0.302, 0.371)	<.0001
rh lag-2-week	medium	0.451	(0.366, 0.535)	1.57	(1.44, 1.71)	<.0001
rh lag-2-week	high	0.808	(0.723, 0.894)	2.24	(2.06, 2.44)	<.0001
**Model-AI**						
AI lag-2-week	medium+high	0.619	(0.549, 0.689)	1.86	(1.73, 1.99)	<.0001
rain lag-2-week	medium	-0.13	(-0.211, -0.048)	0.389	(0.351, 0.433)	0.002
rain lag-2-week	high	-0.943	(-1.05, -0.838)	0.878	(0.81, 0.953)	<.0001
rain lag-1-month	medium	0.401	(0.326, 0.476)	1.49	(1.39, 1.61)	<.0001
rain lag-1-month	high	0.316	(0.221, 0.41)	1.37	(1.25, 1.51)	<.0001
temp lag-2-week	medium	-0.471	(-0.559, -0.383)	0.625	(0.572, 0.625)	<.0001
temp lag-2-week	high	-1.002	(-1.1, -0.9)	0.367	(0.331, 0.406)	<.0001
rh lag-2-week	medium	0.46	(0.378, 0.543)	1.59	(1.46, 1.72)	<.0001
rh lag-2-week	high	1.18	(1.1, 1.26)	3.25	(2.99, 3.54)	<.0001
**Model-HI**						
HI lag-1-month	medium+high	0.807	(0.732, 0.882)	2.24	(2.08, 2.42)	<.0001
rain lag-2-week	medium	-0.982	(-1.09, -0.878)	0.374	(0.337, 0.416)	<.0001
rain lag-2-week	high	-0.142	(-0.223, -0.061)	0.868	(0.8, 0.941)	0.0006
rain lag-1-month	medium	0.206	(0.129, 0.283)	1.23	(1.14, 1.33)	<.0001
rain lag-1-month	high	0.085	(-0.012, 0.183)	1.09	(0.988, 1.2)	0.087
temp lag-2-week	medium	-0.384	(-0.473, -0.295)	0.681	(0.623, 0.744)	<.0001
temp lag-2-week	high	-1.06	(-1.16, -0.954)	0.348	(0.314, 0.385)	<.0001
rh lag-2-week	medium	0.413	(0.33, 0.496)	1.51	(1.39, 1.64)	<.0001
rh lag-2-week	high	1.09	(1.003, 1.17)	2.97	(2.73, 3.23)	<.0001

Next, in order to establish the threshold for entomological surveillance indices as an early warning tool for dengue epidemics, a threshold, where good sensitivity and specificity both reach above 80%, was selected. A threshold with 100% sensitivity but poor specificity will lead to too many false alarms and exhaust public health resources. Therefore, we applied each selected VI models to multiple logistic regression models to examine the accuracy of predicting the occurrence of dengue cases based on the ROC analysis by selecting an operating point which provided an optimum tradeoff between false-positive and false-negative results. The results suggested that Model-AI, BI, CI and HI, based on the operating point selected, yielded a sensitivity of 82, 87, 86 and 85%, respectively; and a specificity of 76, 80, 80 and 80%, respectively (Table 3). The accuracy of Model-AI, BI, CI and HI in predicting the occurrence of dengue cases were 83.8, 87.8, 88.3 and 88.4%, respectively (S2 Fig). The individual predicting thresholds for Model-AI, BI, CI and HI were 0.97, 1.16, 1.79 and 0.997, respectively as shown below. Each of them when combined with meteorological factors had better performance compared to the prediction using AI, BI, CI and HI alone, where the value were only 69.2, 78.7, 80.2 and 78.7% accurate, respectively (Table 3).

$$\begin{array}{l} \text{Model BI}=3.04BI-0.357RF_{11}-1.49RF_{12}-0.096RF_{21}+0.405RF_{22}-1.5Temp_{1}-0.888Temp_{2}\\ \;+0.634RH_{1}+2.77RH_{2}>1.16\\ \text{Model AI}=2.48AI-0.385RF_{11}-1.68RF_{12}+0.719RF_{21}+1.47RF_{22}-1.78Temp_{1}-0.619Temp_{2}\\ \;+1.02RH_{1}+3.36RH_{2}>1.79\\ \text{Model CI}=3.39CI-0.462RF_{11}-1.37RF_{12}+0.013RF_{21}+0.329RF_{22}-1.84Temp_{1}-1.54Temp_{2}\\ \;+0.58RH_{1}+2.61RH_{2}>0.97\\ \text{Model HI}=3.44HI-0.596RF_{11}-2.01RF_{12}-0.337RF_{21}+0.127RF_{22}-1.78Temp_{1}-0.866Temp_{2}\\ \;+0.738RH_{1}+3.12RH_{2}>0.997 \end{array}$$

where the variables definition are the same as Poisson regression variables.

**Table 3:** Prediction accuracy of different mosquito indices by univariate and multivariate logistic regressions.

	univariate				multivariate			
Model	AI	BI	CI	HI	AI	BI	CI	HI
AIC	8447	8231	7373	8360	6801	6524	5647	6638
AUC	0.692	0.787	0.802	0.787	0.838	0.878	0.883	0.884
Sensitivity	0.78	0.84	0.85	0.84	0.82	0.87	0.86	0.85
Specificity	0.6	0.73	0.75	0.73	0.76	0.8	0.8	0.8

### Sensitivity analysis

The estimates of AUCs, as obtained by leave-one-out cross-validation for Model-AI, Model-BI, Model-CI, and Model-HI, were 0.762, 0.818, 0.833, and 0.829, respectively; those by leave-one-year-out were 0.814, 0.85, 0.866 and 0.843, respectively, and only slightly less (2~4%) than the original AUC. The results suggest that our method is stable in predictive accuracy.

## Discussion

With the continuously high levels of worldwide dengue transmission, predicting dengue outbreaks in advance of their occurrence or establishing an early warning system through the combination of climate, environmental, host and vector-based data is of critical importance. The main purpose of an early warning system is the collection of information leading to timely decision making process, which triggers intervention strategies in order to reduce the burden and effect of the disease or outbreak on a specified population. Although mosquito vector is directly involved in virus transmission, the current entomological indicators do not reliably assess the risk of dengue case occurrence. Our study here provided the proof-of-concept results, utilizing a two-stage model to identify the best set of lag effects of meteorological and entomological variables, explaining dengue epidemics based on the data obtained from Taiwan, which is a dengue-non-endemic country. AI, BI, CI and HI of the vector indices when combined with the meteorological factors have better performances compared to the prediction using AI, BI, CI and HI alone, with 83.8, 87.8, 88.3 and 88.4% accuracy, respectively. The advantage of this two-stage model is not only to produce the unified set of predictors throughout two-stage modeling but also to keep as much information in the set as possible. Although the error between the observed and estimated counts could be large, the prediction of peaks by the co-variables selected from the Poisson models quite coincided (S1 Fig). Further employing these co-variables in the second-stage logistic models for predicting the occurrence of outbreak came out with satisfactory results. Since same co-variables were employed in the two-stage model, the value above the threshold would not only predict the occurrence of dengue cases, but also the size of the outbreak based on the stage 1 model, either big or small. Therefore, each country should consider its own individual data and apply this two-stage modeling strategy to find the optimal predictive threshold for allocating public health resources and prevention strategies.

Since only adult female *Aedes* mosquitoes are directly involved in dengue transmission, directly counting dengue vectors (adult female *A*. *aegypti* and occasionally *A*. *albopictus* mosquitoes) using fixed-position traps has been advocated to replace the traditional methods, because *stegomyia* indices are developed many decades earlier for yellow fever and the relationship with dengue transmission is usually ambiguous[39]. However, the *stegomyia* indices such as HI and BI remain central and are most widely used in the monitoring of dengue vector populations, but their critical threshold has never been determined for dengue virus transmission[40,41]. Traditionally, BI < 5 was proposed to prevent yellow fever transmission and three different risks of HI, with <0.1% as low, 0.1–5% as medium and >5% as high, were suggested by the Pan American Health Organization to prevent dengue transmission[42]. However, dengue transmission was observed with vector density below that and the appropriated entomologic level remains contentious[43]. A universal critical threshold applicable across many contexts has never been determined even though a simple threshold (HI = 1% or BI = 5) has been used for many years and is only valid in some situations[44]. Since the population of mosquito vectors is influenced by the meteorological factors, a threshold combining VI and meteorological variables with different lag effects would provide a better prediction of dengue epidemic. In this study, four VI models were developed and integrated thresholds were estimated from the multivariate Poisson model with BI, CI, AI and HI of 1.16, 1.79, 0.97 and 0.997, respectively. These integrated VI thresholds predicted better with accuracy higher than 80%, compared to using VI alone (Table 3). Furthermore, although choosing an arbitrary threshold of BI > 5 is more intuitive and interpretable, the prediction accuracy of dengue epidemic is only 77% in this study. The utilization of single global values of BI or other VI as thresholds for dengue transmission is unreliable and is not recommended based on the previous review[13,37,45]. Therefore, our study utilized a two-stage modeling, which is a simple and direct concept for estimating the thresholds in different locations or counties. An automatic smartphone application which uses the two-stage model to calculate the integrated VI thresholds from the collected data on a weekly basis would facilitate an early warning system for worldwide use.

The meteorological factors (temperature, rainfall and relative humidity) were important variables which directly and indirectly affect the mosquito density and blood feeding behavior[46,47]. Overall, temperature affects the length of *Aedes* gonotrophic cycle, pupae development period and extrinsic incubation period of dengue virus, which are usually shorter at higher temperature[48–50]. Temperatures may also influence the vector body size and its biting behavior. Smaller mosquitoes feed more often than larger ones; and higher temperatures can augment immature development resulting in smaller mosquitoes[51]. Higher temperatures also speed blood meal digestion so that females need to feed more often[52]. Thus, all these factors directly and indirectly influence the contact rate between vectors, which leads to an increased risk of viral transmission from an infected mosquito to a susceptible host[3]. On the contrary, the effect of temperature on the mortality rate of larvae, pupae and adult mosquitoes can be U-shaped with a lower mortality rate seen when temperature ranged from 15 to 30^°^C[53,54]. This might explain the results in our study that showed a positive association of temperature at medium level at 1-month-lag effect with the risk of an increased number of dengue cases, but a negative association with the risk of an increased number of dengue cases at either 1-month lag only at a high level of temperature or 2-week lag, either in medium or high levels of temperature. Non-linear effect of the co-variables on the number of dengue cases could also be found at rainfall, which was also found in our and other’s studies. An increase in amount of rainfall leads to more breeding sites, which in turn lead to an increase in the number of mosquito density as suggested by previous studies[11]. However, too much rainfall might wash away the larvae or pupae inside the premise and decrease the mosquito density[55]. Adding quadratic term is one way to cope with the problem. However, due to the co-linearity between the linear and quadratic term, very few covariates would be significant. Another approach to cope with the nonlinearity is to trisect the covariate (low, middle and high); and we found it can come out with a more significant result for interpretation as shown in this current study.

Other well-known factors may have contributed to the dynamic occurrence of dengue cases and epidemic. The shift of age structure from children to young adults during epidemics was previously reported[56]. The average age in confirmed dengue cases was 44.4 years old and slightly increased from 2005 to 2012, which was consistent with the trend of gradual increase in age from the general population in Kaohsiung city. The slight increase of age in dengue cases was not significantly correlated with the annual dengue incidence rate (P = 0.261) (S3 Fig). Although the ratio of primary and secondary infections might change the epidemic dynamics and increase the disease severity, previous studies found the DHF/DF ratio increased through the epidemic and the disease severity was not correlated with the secondary infection in Taiwan[57]. Previous studies also suggested that certain strain or serotype of DENV with epidemic potential might increase viral growth in mosquito and enhance virus transmission[58,59]. Since not all the confirmed cases were determined by virus isolation or RT-PCR in this study, it was currently not feasible to incorporate the case ratio infected by different serotypes each year into the model. Additionally, herd immunity might affect the dengue epidemic as suggested in other studies but the results were not conclusive[45]. How the herd immunity, measured from the sero-prevalence data which is not available in this study, affects our model prediction requires further study.

The results in this study should be interpreted within the context of strengths and limitations. First, entomologic data collected through routine systems could pose some limitations due to different vector control technicians for inspection, procedures that are not completely uniform and inspection cycles. We focused on high risk areas and inspected the premises for mosquito breeding sites on a weekly basis to minimize the bias. Second, the overall indices were calculated for communities defined by administrative boundaries, which do not constitute entomologically homogeneous units. The optimal geographical level for calculation would be under household and neighborhood level, which is usually difficult to obtain due to the protection of individual privacy. The consistent collection of vector indices under the same administrative boundaries available to be used for public domain would provide better predictions in the long term. Third, the surveillance and dengue case ascertainment did not allow us to detect asymptomatic infection, which likely varied through time and was underestimated in this study. Fourth, the present study was an ecological investigation; therefore, it is not possible to make inferences concerning the causative relationship between the mosquito larvae indices and dengue infection at the individual patient level. Fifth, the spatial heterogeneity was not considered in this study and will be the future focus for developing a better model[60,61]. Sixth, in this study we focused on the high risk district where 97.9% dengue cases occurred and inspection was carried out on a weekly. The potential bias is minimal since the timing of mosquito collection did not depend on the onset of dengue cases and the mosquito collection was not only done in the residential districts of the confirmed dengue cases. However, the threshold estimated in this study could only be applied to the high risk district. If the threshold is desired to be determined in the middle or low risk area, different lag effects of meteorological variables and monthly values of VIs would need to be determined separately. Seventh, when the case was confirmed, the environmental interventions carried out by the health services team would be implemented such as the chemical treatment of the location and the neighborhood of the confirmed case, the intensification of measures to control breeding areas and health education. These usually lead to the elimination of breeding grounds of immature and adult mosquitoes. Since our study was to establish a threshold for early case detection before any control measures is in place, the effect on our model prediction of the occurrence of dengue cases would be minimal.

In conclusion, our study here provided the proof-of-concept of how to search for the optimal model and determine the threshold for dengue epidemics. Unlike other studies with a determined threshold, here the findings cannot be extrapolated to communities with different environment conditions or herd immunity levels. We currently are developing an automatic system allowing implementation of the data in a weekly basis and following the two-stage model to calculate the integrated VI threshold for worldwide use. This work provides an example of the practical utility of research projects in the operational public health field and reinforces the need for a multidisciplinary approach in the understanding and management of vector-borne diseases.

## Supporting Information

S1 Text Additional analysis of the meteorological variables on the dengue case counts from 2005 to 2012. (DOCX)

S1 Fig Secular trend of all laboratory-confirmed indigenous dengue cases from 2005 to 2012 detected by active (solid line) or passive (dash line) surveillance system, accordingly. (TIF)

S2 Fig Receiver operating characteristic (ROC) curve analysis for each vector index. Each vector index model including Model-BI: Breteau index model (A), Model-CI: Container index model (B), Model-HI: House index model (C) and Model-AI: adult *A*. *aegypti* index model (D) generated one ROC curve and the area under the ROC curve was calculated to evaluate the prediction accuracy.
(TIF)

S3 Fig (A) Secular trend of average age of confirmed dengue cases and percentage of general population aged > 40 in Kaohsiung City from 2005 to 2012. (B) Scatter plot of average age of confirmed dengue cases and incidence rate of dengue in Kaohsiung City from 2005 to 2012. (TIF)
