# Supplementary material for: Re-assess Vector Indices Threshold as an Early Warning Tool for Predicting Dengue Epidemic in a Dengue Non-endemic Country
Source: PLoS Negl Trop Dis. 2015 Sep 14;9(9):e0004043. doi: 10.1371/journal.pntd.0004043 (PMC4569482; doi:10.1371/journal.pntd.0004043)
Supplement: S1 Text — (DOCX) [file pntd.0004043.s001.docx]

The histogram distribution of the meteorological variables on the dengue case counts was shown in the figures below. The red dots and blue triangles indicated the breaks of 33th and 67th percentiles used to transform the data into three levels: low, medium and high. The nonlinear effect of the meteorological variables on the dengue case counts was noted. Similar results were also found using the boxplots on three different levels of meteorological variables. Furthermore, the univariate results from Poisson regression models using continuous predictors with a quadratic term suggests that the number of DF cases is a quasi-concave function of the 1-month-lag rainfall, which resembles the trisection results of in both univariate (Table 1) and multivariate (Table 2), with low rainfall correspond to fewest DF cases, middle rainfall correspond to most DF cases, and high rainfall correspond to moderate DF cases. On the other hand, the number of DF cases is a decreasing function of 2-week-lag rainfall, which resembles the trisection results in multivariate only. Due to the nonlinear effect of the meteorological variables on the dengue case counts and the trisection results are similar to continuous results with quadratic function, trisection variables were used in the following two-stage modeling for intuitively understanding.

1.) Histograms of the meteorological variables giving the breaks used to define the low, medium, and high bins. The red dots and blue triangles indicated the breaks of 33th and 67th percentiles used to transform the data into three levels.


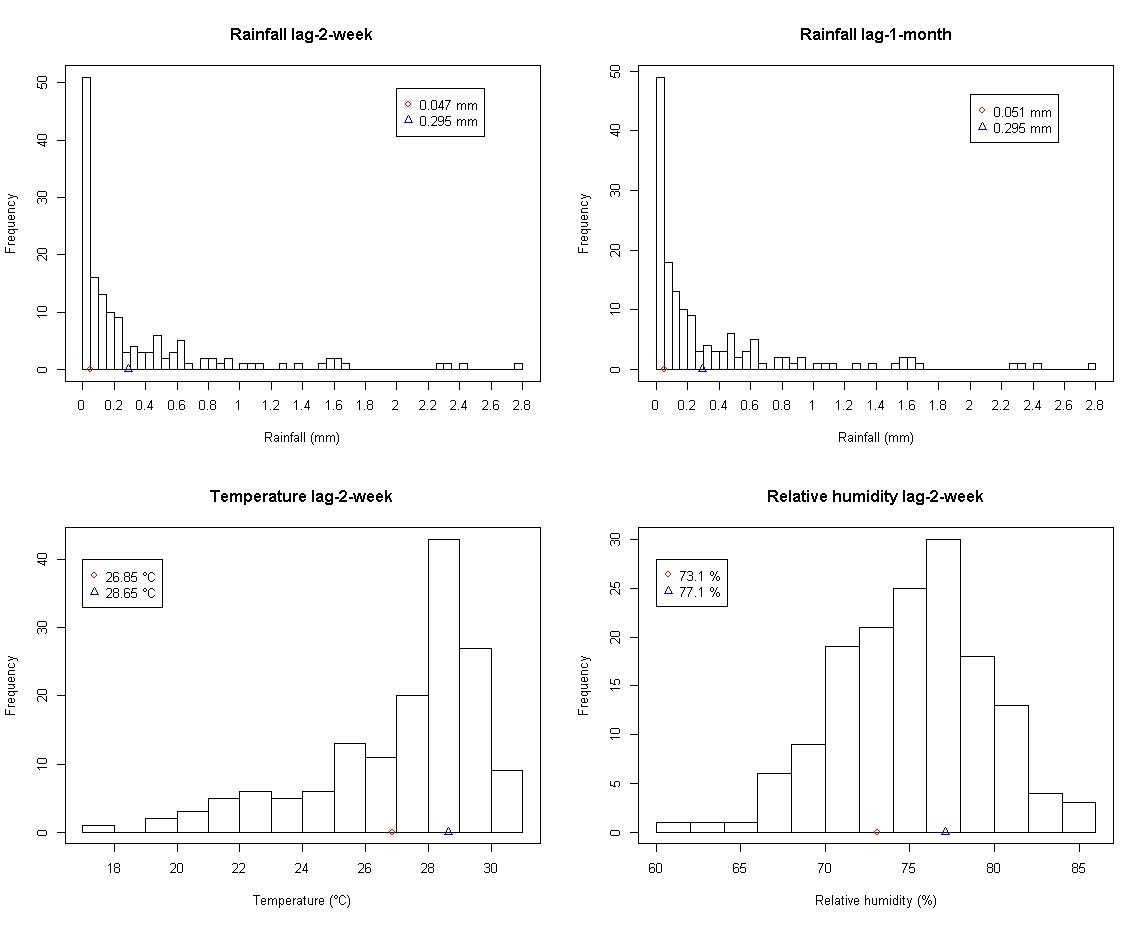


2.) Boxplots of dengue cases against three different levels of meteorological data


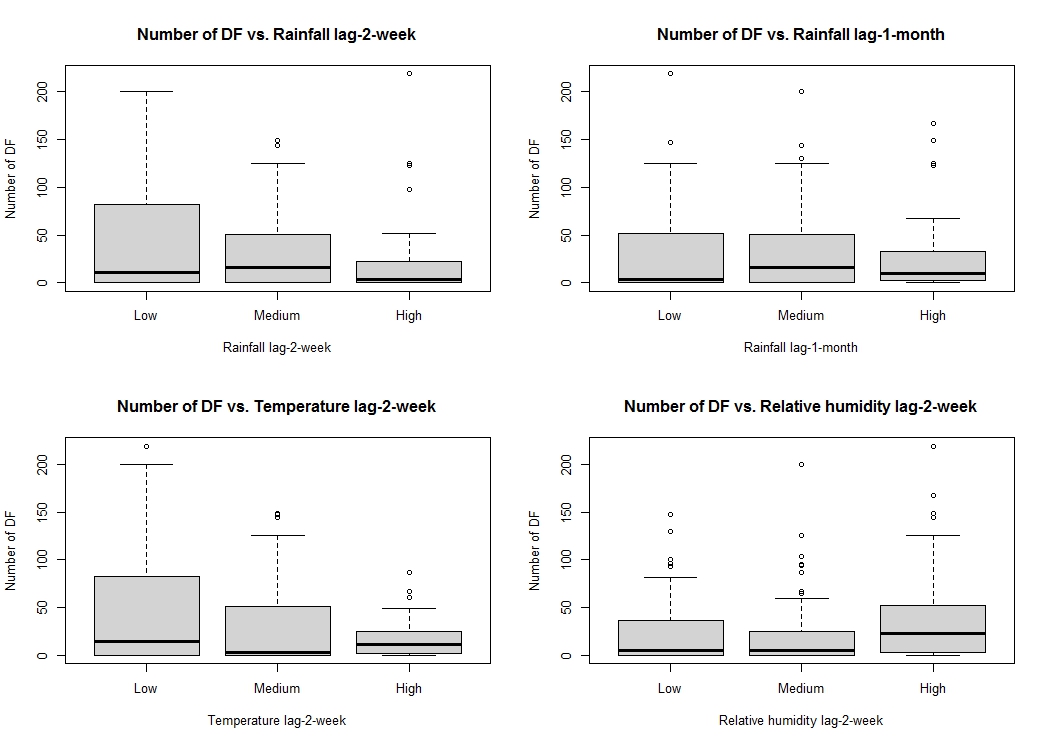


3.) Univariate results from regression using standardized continuous predictors with a quadratic term.

|  | estimator | standard error | p-value |
| --- | --- | --- | --- |
| rainfall lag-2-week | -0.624 | 0.06 | <.0001 |
| rainfall lag-2-week (quadratic term) | 0.172 | 0.043 | <.0001 |
| rainfall lag-1-month | 0.113 | 0.055 | 0.042 |
| rainfall lag-1-month (quadratic term) | -0.235 | 0.044 | <.0001 |
| temperature lag-2-week | -0.209 | 0.009 | <.0001 |
| temperature lag-2-week (quadratic term) | -0.056 | 0.002 | <.0001 |
| relative humidity lag-2-week | 0.048 | 0.004 | <.0001 |
| relative humidity lag-2-week (quadratic term) | -0.0003 | 0.001 | 0.625 |
