# Supplementary figures and images for: Re-assess Vector Indices Threshold as an Early Warning Tool for Predicting Dengue Epidemic in a Dengue Non-endemic Country
Source: PLoS Negl Trop Dis. 2015 Sep 14;9(9):e0004043. doi: 10.1371/journal.pntd.0004043 (PMC4569482; doi:10.1371/journal.pntd.0004043)

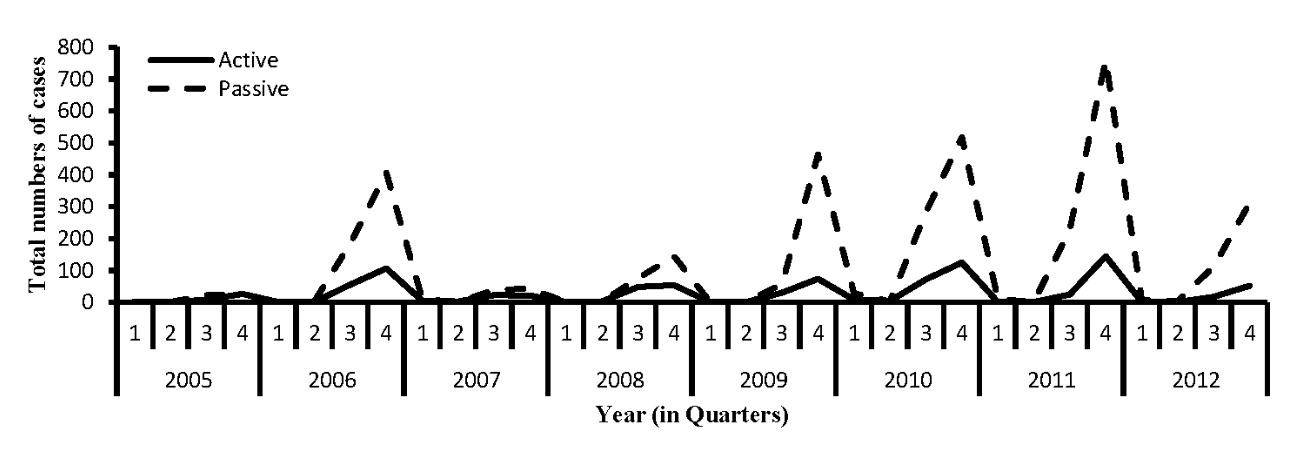

Supplement: S1 Fig — (TIF) [file pntd.0004043.s002.tif]

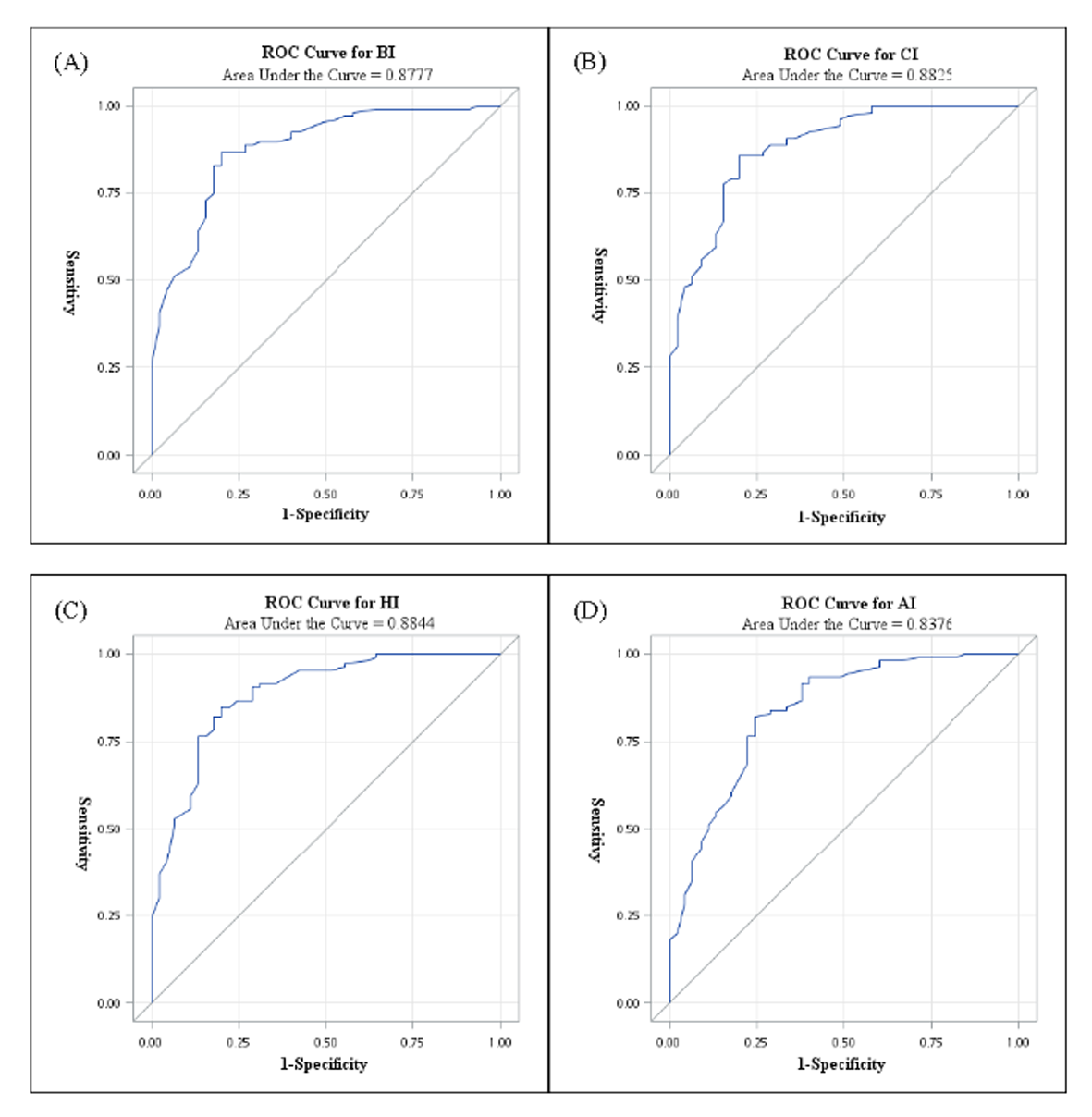

Supplement: S2 Fig — Each vector index model including Model-BI: Breteau index model (A), Model-CI: Container index model (B), Model-HI: House index model (C) and Model-AI: adult A. aegypti index model (D) generated one ROC curve and the area under the ROC curve was calculated to evaluate the prediction accuracy. (TIF) [file pntd.0004043.s003.tif]

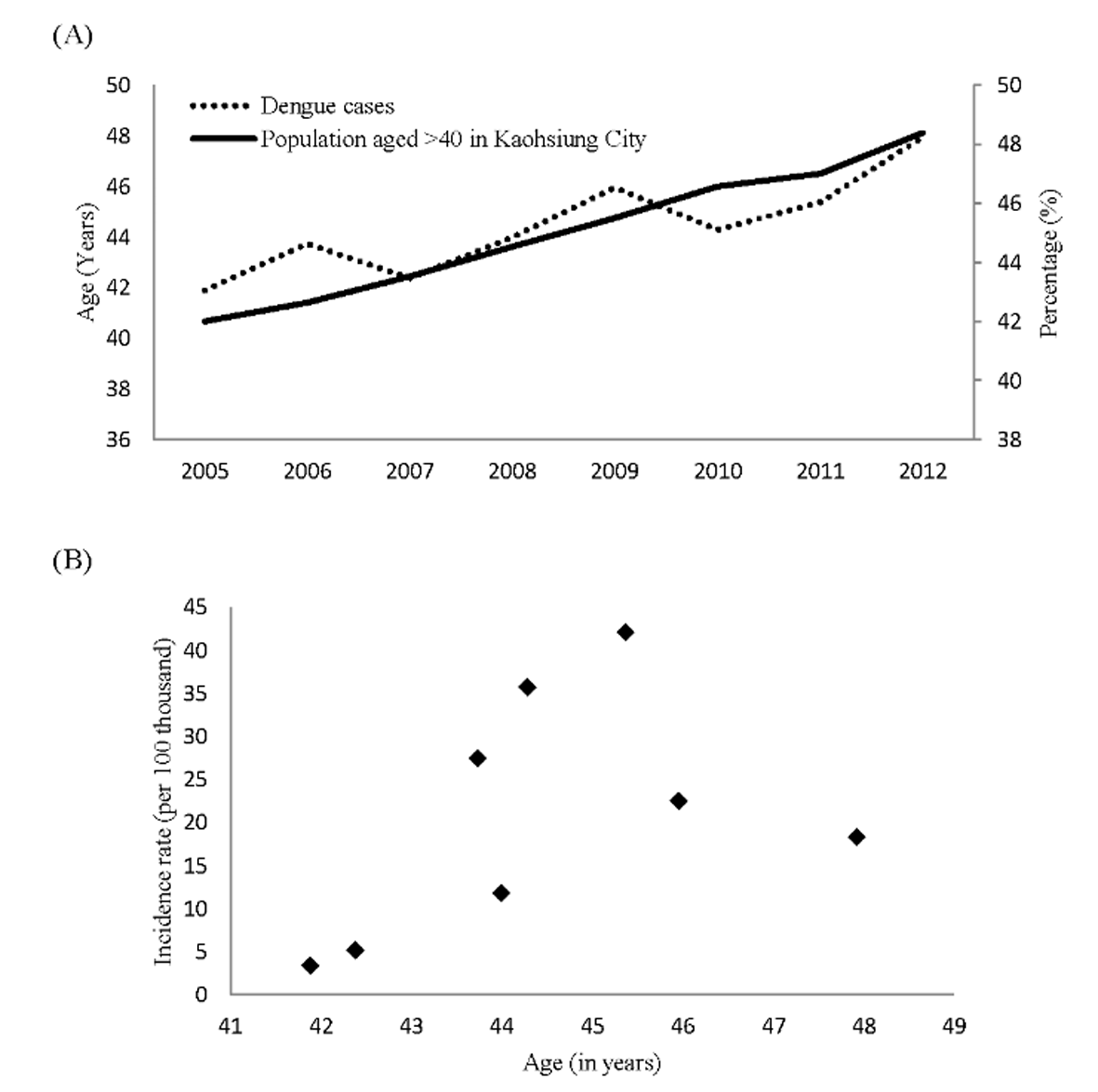

Supplement: S3 Fig — (TIF) [file pntd.0004043.s004.tif]
